# BreedVision — A Multi-Sensor Platform for Non-Destructive Field-Based Phenotyping in Plant Breeding

**DOI:** 10.3390/s130302830

**Published:** 2013-02-27

**Authors:** Lucas Busemeyer, Daniel Mentrup, Kim Möller, Erik Wunder, Katharina Alheit, Volker Hahn, Hans Peter Maurer, Jochen C. Reif, Tobias Würschum, Joachim Müller, Florian Rahe, Arno Ruckelshausen

**Affiliations:** 1 Competence Centre of Applied Agricultural Engineering (COALA), University of Applied Sciences Osnabrück, 49076 Osnabrueck, Germany; E-Mails: L.Busemeyer@hs-osnabrueck.de (L.B.); d.mentrup@hs-osnabrueck.de (D.M.); K.Moeller@hs-osnabrueck.de (K.M.); Erik.Wunder@hs-osnabrueck.de (E.W.); 2 State Plant Breeding Institute, Universität Hohenheim, 70593 Stuttgart, Germany; E-Mails: Katharina.Alheit@uni-hohenheim.de (K.A.); Volker.Hahn@uni-hohenheim.de (V.H.); hpmaurer@uni-hohenheim.de (H.P.M.); Jochen.Reif@uni-hohenheim.de (J.C.R.); Tobias.Wuerschum@uni-hohenheim.de (T.W.); 3 Institute of Agricultural Engineering, Universität Hohenheim, 70593 Stuttgart, Germany; E-Mail: Joachim.Mueller@uni-hohenheim.de; 4 AMAZONEN-Werke H. Dreyer GmbH & Co. KG, 49205 Hasbergen-Gaste, Germany; E-Mail: dr.florian.rahe@amazone.de

**Keywords:** field trials, plant phenotyping, multi-sensor fusion, image-based sensors, plant breeding, sensor platform

## Abstract

To achieve the food and energy security of an increasing World population likely to exceed nine billion by 2050 represents a major challenge for plant breeding. Our ability to measure traits under field conditions has improved little over the last decades and currently constitutes a major bottleneck in crop improvement. This work describes the development of a tractor-pulled multi-sensor phenotyping platform for small grain cereals with a focus on the technological development of the system. Various optical sensors like light curtain imaging, 3D Time-of-Flight cameras, laser distance sensors, hyperspectral imaging as well as color imaging are integrated into the system to collect spectral and morphological information of the plants. The study specifies: the mechanical design, the system architecture for data collection and data processing, the phenotyping procedure of the integrated system, results from field trials for data quality evaluation, as well as calibration results for plant height determination as a quantified example for a platform application. Repeated measurements were taken at three developmental stages of the plants in the years 2011 and 2012 employing triticale (×*Triticosecale* Wittmack L.) as a model species. The technical repeatability of measurement results was high for nearly all different types of sensors which confirmed the high suitability of the platform under field conditions. The developed platform constitutes a robust basis for the development and calibration of further sensor and multi-sensor fusion models to measure various agronomic traits like plant moisture content, lodging, tiller density or biomass yield, and thus, represents a major step towards widening the bottleneck of non-destructive phenotyping for crop improvement and plant genetic studies.

## 1. Introduction

The constantly increasing World population, likely to exceed nine billion by 2050, has led to a growing demand for agricultural products [1]. To achieve food and energy security for the future is one of the major challenges for plant science and crop improvement in the 21st century. The development of improved crop varieties depends on our ability to assess the phenotype of plants in the field. Most of the currently available phenotyping methods are, however, labor and time consuming, not totally objective or destructive [2,3]. Thus, measurements are limited with regard to the number of plots that can be evaluated and with regard to the traits that can be assessed. This lack of suitable phenotyping capabilities is now recognized as a major factor limiting the development of improved crop varieties [4–6]. Consequently, there is an urgent need for the development of novel methods in phenomics for a non-destructive determination of diverse traits under field conditions [2,7]. In comparison to traditional phenotyping, novel approaches will have to enable the screening of a higher number of plants per time, or provide information on phenotypes that so far have been impossible to measure in routine plant breeding (e.g., response to abiotic stress, growth dynamics, or primary and secondary metabolites).

Several attempts have been made based on single sensors to measure plant parameters using laser distance sensors, laser and ultrasonic rangefinders, light curtains, spectrometers, digital cameras or radar. For example laser distance sensors were used to determine plant height and crop density [8–10], laser rangefinders were applied for the measurement of plant height and biomass [11] as well as for determining crop density [12], light curtains were used to measure plant height [10,13,14], spectrometers were applied to measure the nitrogen status [15] and to determine crop yield [15–17] and digital cameras were used to determine the leaf area index (LAI) and grain yield of wheat [18]. In most cases the above mentioned methods were based on determination of plant parameters using only one type of sensor and one single parameter calculated from sensor raw data. Using single sensors enables measuring simple traits like plant height, but from a technical point of view shows constraints in the determination accuracy of more complex traits like biomass yield. To overcome this limitation, the concept of sensor and data fusion has been suggested to enhance the quality of phenotypic measurements for complex traits [19].

For measurements under field conditions, the combined use of light curtains, CMOS-cameras, triangulation and ultrasonic as well as pressure sensors have been used for plant/weed detection [20]. Biomass of maize at an early stage of development has been measured using light-curtains and spectral reflectance sensors [14]. However, multi-sensor platforms to measure multiple traits and the use of sensor fusion to determine complex traits are still lacking for small grain cereals.

The focus of this paper is on the technological development of a multi-senor phenotyping platform for small grain cereals that delivers a robust basis for non-destructive measurements of diverse traits. In detail, the objectives of this study were: (1) to develop a mobile multi-sensor phenotyping platform for small grain cereals under field conditions, including the mechanical construction and the hard- and software development for multi-sensor data collection; (2) to implement an applicable phenotyping procedure for data collection and analysis and (3), to evaluate the data quality generated with the phenotyping platform.

## 2. Design of the “BreedVision” Phenotyping Platform

### 2.1. Mechanical Concept

**Figure 1.**
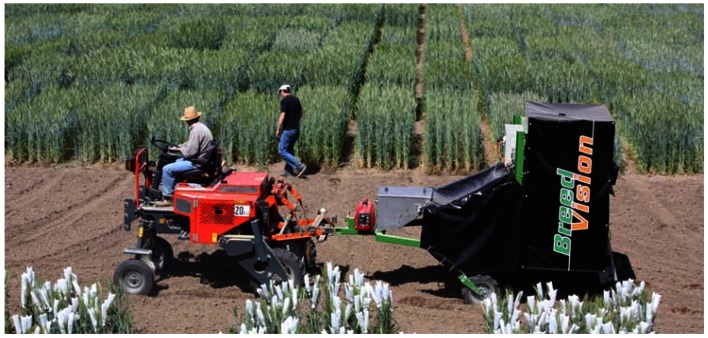
Sensor platform during outdoor measurements in the field.

The mechanical design of the phenotyping platform is illustrated in Figure 1, which shows the platform during field measurements. The system is a tractor pulled trailer with a track width of 1.25 m designed for non-invasive phenotyping of plant breeding trails of small grain cereals up to a plant height of 1.6 m. The phenotyping platform is composed of a carrier vehicle and a sensor module. The sensor module incorporates all sensor systems and the corresponding electronic devices for data collection. It is mounted at the rear of the carrier vehicle and shaded with a black canvas to avoid exposure to direct solar irradiation. The construction of the carrier vehicle enables an electrically powered height adjustment of the module to adapt the platform to different operating situations.

### 2.2. Power Supply

The system has its own uninterrupted power supply integrated in front of the trailer. It consists of a generator with 230 V alternating current with a maximum power output of 0.8 kW driving an AC/DC battery charger (12 V, 50 A) which is connected to an Absorbent Glass Mat (AGM) battery (12 V, 240 Ah). Different power supply units connected to the battery deliver different voltage levels (5 V, 8 V, 12 V and 24 V) to supply the sensor systems and electronic devices with electric energy.

### 2.3. Sensor Systems

The non-destructive, simultaneous determination of different crop parameters like plant height, plant moisture content, tiller density and dry biomass yield based on data collected from a single pass of the field plots requires multiple and different types of sensors. Whereas plant height can be measured with a single sensor, for example with a laser distance sensor measuring from top view into the plants or with a vertical light curtain penetrating the canopy, more complex traits like for example dry biomass yield require multi-sensor fusion concepts to achieve accurate results [19]. Dry biomass yield is influenced by the volume of the plants which itself is affected by the number and the habitus of the plants. Beside the required volume estimation the second important parameter for dry biomass determination is the moisture content of the plants. To be able to collect these different information, various optical sensors suitable for outdoor measurement conditions have been selected and integrated into the system. The sensors have different and to some extent complementary selectivities to morphological and spectral properties of the plants which enables multi-sensor fusion to determine complex traits, increases the robustness of the system and enables multiple trait determination. In detail, it is equipped with two 3D Time-of-Flight (ToF) cameras, a color camera, three laser distance sensors (LDS), a hyperspectral imaging (HSI) system and two light curtain (LC) imaging systems for phenotyping, a webcam for additional measurement documentation, as well as an incremental rotary encoder with a resolution of 1 mm (related to the distance to the ground) and GPS receiver for positioning. The technical details (Table 1) and description of the sensors are as follows:

*Laser distance sensors (LDS):* Both types of LDS integrated into the platform are based on triangulation principle and are characterized by point-wise measurements with high frame rate. Due to this point-wise measurement principle this type of sensor delivers data with a high resolution of a small section of the plants and has selectivity to plant height and to tiller density [8–10].
*3D Time-of-Flight (ToF) cameras:* 3D ToF cameras directly deliver three-dimensional information about plants. In comparison to other techniques like stereo imaging, laser line methods or laser scanners further reconstructions are not necessary which reduce the complexity of the generated data sets. The distance to the object for every pixel of the camera is calculated by ToF measurement of the emitted modulated light (20 MHz). The cameras have a resolution of 50 × 64 pixels and are suitable for dynamic outdoor plant phenotyping [21].
*Light curtain (LC) imaging:* A LC is a linear set of stacked light barriers. Moving LCs along the plots and putting the scanned vertical lines in a consecutive order results in a binary image of the measured object in side view. The LCs consist of 288 light barriers each with an interspacing of 2.5 mm and a measurement frequency of 170 Hz which results in a resolution of 2.5 mm in the vertical and 3 mm in the moving direction of the platform, assuming an operating speed of 0.5 ms^−1^. Although the resolution of LC imaging is lower than the resolution of images collected with a digital camera, further segmentation steps to identify pixels belonging to plants with all its potential errors becomes superfluous, which makes the LC imaging technique applicable for outdoor plant phenotyping. The system is equipped with two LCs, a lower and an upper LC, mounted one upon the other to double the measurement range from 718 mm to 1,436 mm. The bottom edge of the lower LC is located about 200 mm above ground level which allows the monitoring of plants with heights up to 1.6 m. LCs are suitable to measure plant height [10,13,14] and have selectivity to tiller density which are both relevant traits itself but also important parameters for dry biomass determination. In addition, due to the fact that the output of the LCs can be plotted in binary images of the plants from side view, LC data can be used for field trial documentation and to gather surplus information like lodging or the angle of the spikes.
*Hyperspectral imaging (HSI):* A transmission spectrograph based on a line scan method with a measurement frequency of 100 Hz is used. Every scanned line consists of 252 spatial regions each containing 320 spectral pixels covering a range of wavelengths between 970 nm and 1,670 nm. Direct solar irradiation is excluded by a sunblind and a constant illumination is realized by an active 120 W halogen lighting system. Assuming an operational speed of the platform of 0.5 ms^−1^, hyperspectral images of the plants with a resolution of 3 mm in the transverse and 5 mm in the moving direction are feasible. The HIS system is suitable for *in-situ* plant moisture content determination [22], holds the potential to measure the nitrogen status of the plants and is therefore an important extension of the above described morphologically selective sensors of the platform.

**Figure 2.**
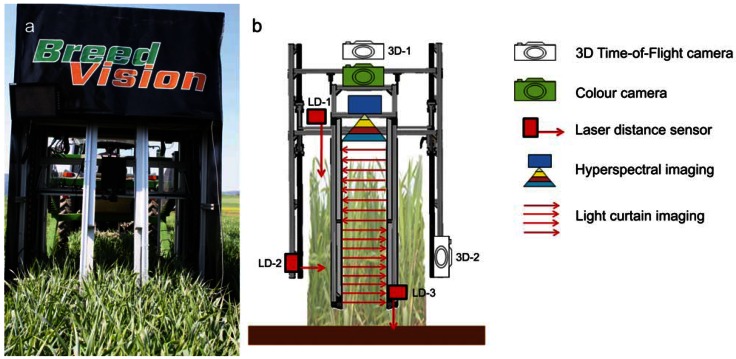
(**a**) Sensor platform from rear view and (**b**) mounting layout of the used sensors.

All sensor systems for the phenotyping process are integrated into the sensor module (Figure 2(a)). The mounting positions of the different types of sensors are illustrated in Figure 2(b). The color camera, one 3D ToF camera (3D-1), HSI and LDS-1 are mounted above plant height for vertical top view measurements of the canopy. To achieve constant distance of about 500 mm to the canopy, even under varying plant heights, the top view sensors are attached to an electrical height adjustment mechanism. The second 3D ToF camera (3D-2) and LDS-2 are measuring the plant cover of the plots in a horizontal side view. The LCs are penetrating the canopy and are reaching the inner rows of a plot. LDS-3 is mounted at the bottom edge of the lower LC to determine the distance to ground.

## 3. Data Management

### 3.1. Data Collection

**Figure 3.**
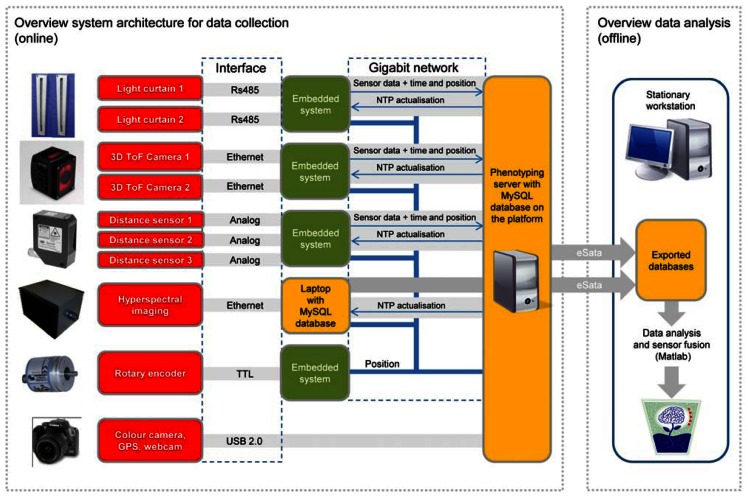
System architecture for data collection and analysis.

The system architecture for data collection is a challenging and important part in the development of multi-sensor phenotyping platforms. The data collection system has to fulfill three major requirements. It has to facilitate: (1) the recording of multiple sensor data with different frame rates, interfaces and data types with the highest possible resolution (2), in a way which offers the opportunity to superpose the datasets in sensor fusion models during the offline data processing process and (3), in addition the system has to be modular to enable the extension of the platform with novel sensors. Figure 3 illustrates the modular system architecture of the platform which was designed as an extended version based on previous work described by Klose *et al.* [23].

The central component of the data collection system is an industrial PC, the so-called phenotyping server, which incorporates a MySQL database server for data storage and a graphical user interface to control the platform. Except for the integration of the colour camera, GPS and webcam which was done by USB 2.0, a gigabit ethernet was chosen as main communication bus between the phenotyping server and the peripherals. To adopt the different communication interfaces of the sensors to ethernet, every sensor is connected to its own microcontroller. Beside this gateway function, the microcontrollers assign a global time (resolution 1 ms) and position (resolution 1 mm) stamp of the beginning and end of the individual data fetch to every measurement value before transmitting it to the phenotyping server. These transmitted datasets are stored in separate database tables for every sensor with the highest possible sensor specific frame rate. This concept ensures a data collection method exploiting the maximum performance of all types of sensors. In addition, the concept allows an extension of the platform with novel sensors without affecting the frame rates of the already integrated systems. However, due to the assigned time and position stamps to the single measurement values and the known offsets between the sensors, it offers the opportunity for sensor-fusion in an offline data analysis procedure which would not be possible with data individually collected for each sensor separately. To ensure precise time information the clocks of all peripherals are synchronized by a NTP-Server that runs on the phenotyping server. To generate position information with adequate spatial resolution, an incremental rotary encoder is mounted at the wheel of the trailer. It has a resolution of 1 mm and sends the actual position stamp via ethernet to all peripherals in the network. Due to high data rates of the HSI system (10 MB/s), the data is stored in an extra MySQL database on a laptop to reduce network traffic in the subnet of the phenotyping server. The global time and position stamp is also transferred to the HSI subnet and is being attached to each HSI dataset directly on the laptop. The databases of the phenotyping server and the HSI laptop can be exported via an eSata interface into a single MySQL database running on a stationary workstation. Data analysis is being performed offline on the workstation using algorithms developed with the software package MATLAB^®^ (The MathWorks^®^, Natick, MA, USA).

### 3.2. Data Processing

Since the collected raw data has no selectivity to any plant parameter itself, offline plant and trait specific data processing strategies are important for a successful phenotyping procedure. Trait specific algorithms are applied to the raw data of the different types of sensors to calculate single parameters for each plot with selectivity to a certain trait or part of the trait. There are two different options to analyze the raw data and to calculate parameters with selectivity to certain plant traits. Sensor models for data reduction may use statistical or image analysis based methods. As an example for statistical data processing methods, the following algorithms were developed focusing on data generation with selectivity to plant biomass of small grain cereals.

Data of the LCs were used to determine: (1) coverage density as percentage of interrupted light barriers of the lower LC, and (2) plant height by determining the average of the 1% highest interrupted light-barriers in combination with the laser distance sensor at the bottom edge of the lower LC which measured the distance of the LC to the ground. A possible influence of low parts of the plants during distance measurements to the ground was excluded by calculating the average of the 3% maximum distances of the collected data. To get more than one average distance value for each plot, the data of every plot was segmented into increments with a length of 10 cm and the average distance to the ground was estimated for each increment.

Data of the laser distance sensors were used to extract information on: (1) plant height as the difference between the average of the 3% maximum distances and the average of 3% minimum distances monitored with the laser distance sensor measuring from top; (2) penetration-depth from top as the difference between the average of all values and the average of the 3% minimum distances of the laser distance sensor measuring from top, and (3), penetration-depth from the side as average of the values measured with the laser distance sensor from the side.

Information monitored with the 3D ToF cameras was used to extract: (1) plant height as the difference between the average of the 3% maximum distances and the average of the 3% minimum distances of all pixels and pictures of a plot from 3D ToF camera measuring from top; (2) penetration-depth from top as the difference between the average of all distances and the average of the 3% minimum distances of all pixels and pictures of a plot, and (3) penetration-depth from the side as the mean distance of all pixels and pictures from 3D ToF camera measuring from the side.

The spatial resolution of the hyperspectral imaging system, enabling plant-soil segmentation, is the basis for precise trait determination based on spectral reflectance data. A first algorithm for plant-soil segmentation based on the spectral angle mapper method was implemented and the coverage density as percentage of detected plants was calculated. All algorithms were developed with the software package MATLAB^®^.

### 3.3. The Phenotyping Process

**Figure 4.**
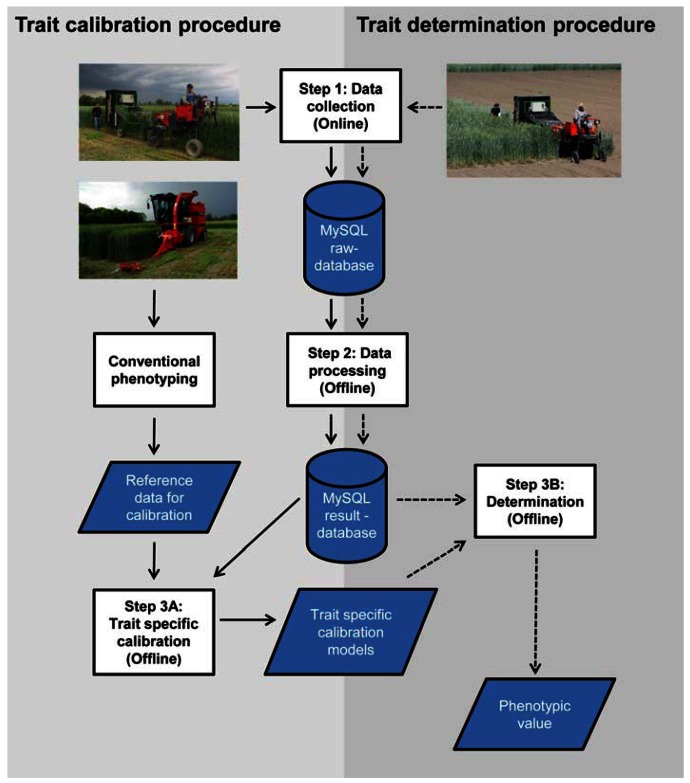
Trait calibration and trait determination procedure, each incorporating the three steps of the developed phenotyping process: Data collection on the field (step 1), data processing on a stationary workstation in the lab (step 2) and trait specific calibration/determination in the lab (step 3A/B).

The phenotyping process is composed of two procedures: A trait calibration and a trait determination procedure with three steps each, illustrated in Figure 4. The first step of both procedures is the raw data collection in the field. At the beginning of a measurement series a field plan defining the plot numbers of all rows in the experiment is loaded into the data collection software. The data collection is manually started at each row in the field by using the graphical interface of the data collection software. Sensor values with corresponding time and position information are stored in separate database tables of the so called MySQL raw-database for each sensor on the phenotyping server. The entire row, normally consisting of 10–30 single plots, is measured and the height of the sensor systems measuring from top view is adapted to the actual height of each plot during the measurement by the operator. At the end of every row the data collection is stopped. The system stores a time and position stamp at every start and end of a row with the corresponding row information defined in the field plan which offers row-wise accessibility of sensor raw data for further data analysis.

**Figure 5.**
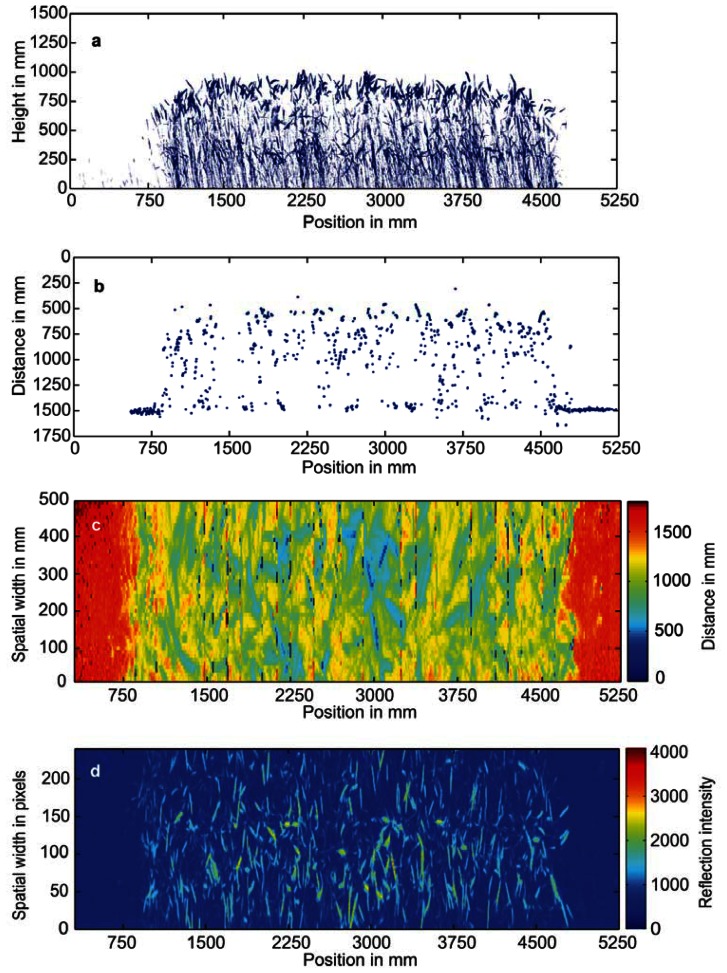
Information gathered per plot for (**a**) light curtains, (**b**) laser distance sensor measuring from top into the plant cover, (**c**) 3D Time-of-Flight camera measuring from top, and (**d**) hyperspectral imaging, (exemplarily shown for 970 nm) measuring from top.

The second step of both procedures is the offline raw data processing on a stationary workstation. An algorithm based on data of the LCs divides the raw data of every row into sections corresponding to the single plots of the row. For each detected plot the algorithm determines the time and position for every start and end of the plot and stores the information in a separate database table. This generated information enables a plot-wise accessibility of the sensor raw data as graphically depicted for a single plot in Figure 5.

Data processing algorithms applied on the raw data lead to different single values for each detected plot which are stored in the so called MySQL result-database. These obtained values can either be used to generate new species and trait specific calibration models or to determine phenotypic values (step 3). Regardless of whether the measurement was performed for trait determination or trait calibration, in both cases steps one and two are identical and only step 3 is different. Whereas the obtained data processing results are used in combination with the conventionally measured traits for trait calibration (step 3A in the trait calibration procedure), trait determination requires data processing results and existing calibration models to calculate the phenotypic trait of interest (step 3B in the trait determination procedure).

### 3.4. System Integration

The phenotyping procedure is a complex process which contains several steps between the data collection process and the output of calculated phenotypic values. From a practical point of view it is essential to combine all single steps into an integrated phenotyping system to achieve a user friendly operation, even for non-expert users. In line with this requirement, integrated software tools with graphical user interface for data collection and data analysis have been developed.

The data collection software provides all important information of the platform and delivers an interface to control the system. For example, it gives information about the status of every sensor, the actual CPU load of the system and remaining free disk space on the phenotyping server. It also provides visualization of the actual sensor values, control buttons to start and stop the measurements, as well as mechanisms to load pre-defined field plans for optimized measurement scheduling. The software for data analysis, running on a stationary workstation in the lab, combines all required steps of the data analysis procedure and was developed with the MATLAB^®^ software package. It automatically detects new measurements in the raw-database as well as missing data processing results caused by an interrupted previous data analysis or new data processing algorithms. It performs all necessary calculations and stores the results in the result-database. New calibrations can be defined and the results of trait determination are stored in Microsoft Excel^®^ files. The software can be extended by adding Excel® files with new calibrations or new algorithms for data processing which are dynamically integrated into the data analysis procedure. This flexible structure enables the adaption of the platform to new traits and different species without changing the entire data analysis structure.

Due to the graphical user interface for data collection, the common file format to interact with the data analysis software and the automated data processing structure, the phenotyping platform is prepared to be used even by non-expert users in the plant breeding industry.

## 4. Application of the Phenotyping Platform

### 4.1. Field Trials for Data Quality Evaluation

**Figure 6.**
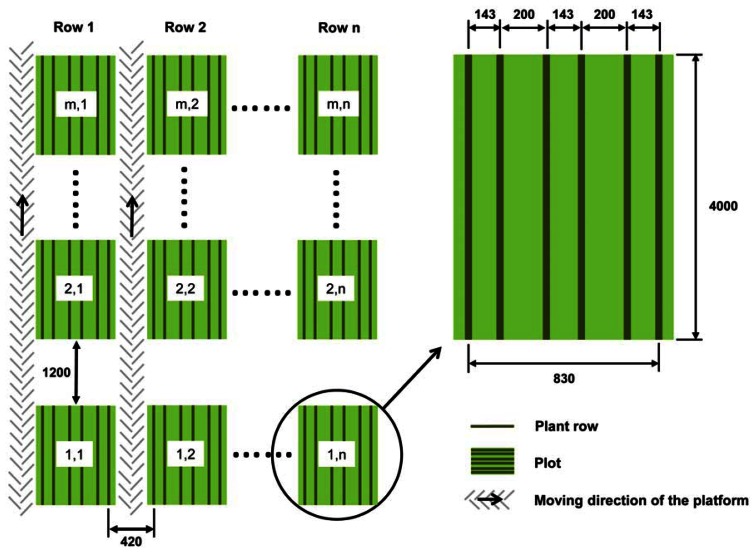
General design of the field trials and moving direction of the platform, the interspaces between the plots and the dimensions of one single yield trial plot. All dimensions are specified in mm, *n* denotes the number of rows (2011: n = 48; 2012: n = 24) and *m* the number of plots per row (2011: m = 14; 2012: m = 25).

Field experiments for data quality evaluation of the phenotyping platform were conducted in Stuttgart, Germany, in the years 2011 and 2012 and were based on 25 diverse genotypes of triticale (×*Triticosecale Wittmack* L.) cultivated in a standard field design for plant breeding trials. The interspacing between the plots, the dimensions of a single plot and the moving direction of the phenotyping platform are illustrated in Figure 6. Every plot consisted of six plant rows with a length of about 4 m and a width of about 1 m. The only modification to a standard experimental field design was the enlarged distance between the second and third and between the fourth and fifth plant row to ensure a non-destructive penetration of the LCs. The field experiments were subdivided into three identical parts, comprising 200 plots each, to generate plant material for sensor calibration and validation at three different growth stages ([24] BBCH 50–59; BBCH 60–69; BBCH 70–79). Every genotype was sown in two plant densities (140 and 280 plants per m^2^) and treated with two nitrogen supply levels (standard practice and 50% reduction) to achieve a high variation of parameters for sensor calibration. The whole experiment was repeated twice. In the year 2011, the experiment consisted of n = 48 rows with m = 14 plots each, in 2012 the experiment consisted of n = 24 rows with m = 25 plots each.

Data was collected with the phenotyping platform in both years at all above mentioned growth stages of the plants. During the measurements the platform was pulled by a tractor with a constant speed of 0.5 ms^−1^ enabling the measurement of up to 2,000 plots per day. Every plot in 2011 and 2012 was recorded twice within a repetition time of less than 10 minutes except for the third growth stage of the year 2011, where the period of time between the repeated measurements was about 90 minutes. The phenotyping of the plots was performed during daytime and under dry weather conditions. In addition, traits like plant height, fresh weight density, moisture content, growth stage, tiller density and nitrogen content of all plots were measured by conventional methods to generate reference data for sensor calibrations.

### 4.2. Quality Evaluation of the Phenotyping Procedure

The quality of the phenotyping procedure depends on the robustness of raw data collection, the ability to map the row-wise collected raw data to the corresponding plots within the rows and the quality of the data processing algorithms. To quantify the performance of the integrated sensor systems and the developed phenotyping procedure in its field of application, the “technical repeatability” of data processing results across repeated measurements of the years 2011 and 2012 was analyzed. The technical repeatability, not to be mixed up with repeated genotypes in field trials for heritability estimation, is an important parameter to characterize the robustness of the sensors itself, as well as, the quality of the data collection and data processing system. The following statistics were applied to describe the technical repeatability of every parameter calculated during the data processing procedure: Mean relative error of repetition:

(1) $${\text{MRE}}_{w}=1/n\cdot \sum_{i=1}^{n}\left|{\hat{y}}_{1i}-{\hat{y}}_{2i}\right|/{\hat{y}}_{1i}$$

where *n* denotes the number of repeated samples in the dataset, *ŷ_1_*_i_ the first and *ŷ_2_*_i_ the second repetition of the same plot and the coefficient of determination of repetition as the squared correlation:

(2) $${R_{w}}^{2}=r^{2}({\hat{y}}_{1i},{\hat{y}}_{2i})$$

**Figure 7.**
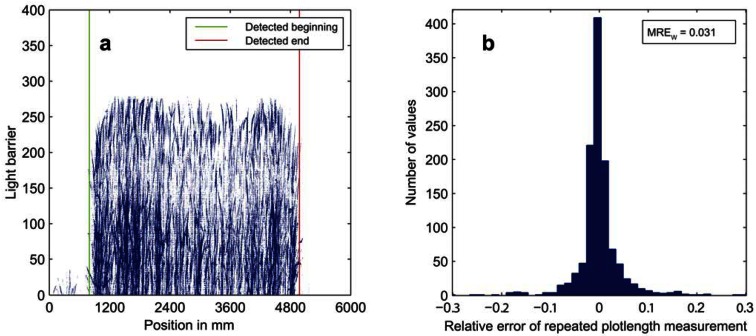
(**a**) Light curtain data with detected start and end of a single plot and (**b**) histogram of relative errors of repeated plot detections during measurements of the years 2011 and 2012 based on two repetitions of 1,200 samples. *MRE_w_* denotes the mean relative error between repeated plot length measurements.

The robustness of mapping the raw data to the corresponding plots has been quantified by determining the repeatability of plot length measurements. Therefore, the output of the plot detecting algorithm was used to estimate the length of the plots as the distance from detected start position to the end position. This way MRE_w_ of repeated plot length measurements from 2011 and 2012 was calculated. The result, summarized in Figure 7, underlines the suitability of the developed data mapping method and offers a cost efficient alternative to high precision GPS methods like Differential Global Positioning System (DGPS) or Real Time Kinematic (RTK).

The second important part to achieve calibrations with high accuracy is the quality of collected raw data and performed data processing results. Therefore, besides the selectivity of the developed algorithms for data processing to the traits of interest, the precision of the different types of reduced data is important. The results of performance evaluation based on data of the years 2011 and 2012, as summarized in Table 2, confirm the applicability of the platform for robust outdoor measurements of small grain cereals.

The repeatability of all data processing results of every sensor is high (R_w_^2^ > 0.6), except for parameter penetration-depth from the side derived from data of the laser distance sensor and data of the 3D ToF camera, both measuring from the side view, for growth stage BBCH 70-79. This decrease in repeatability can be explained by a change of the data collection procedure during harvest 3 in the year 2011. The second repetition of data collection during this measurement was performed in the opposite direction. Due to this change in moving direction of the platform, the sensors measuring from the side view collected data of the left two rows of the plots during repetition 1 and of the right two plant rows of the plots during repetition 2. Disregarding this slight decrease in repeatability, the system delivers high precision phenotypic data which is the basis for the development and calibration of high quality sensor fusion models.

### 4.3. Trait Calibration

Besides the precision of the data collection system and the data processing algorithms, of course the selectivity of the developed data processing algorithms and sensor fusion models to the phenotypic traits of interest are of high importance. The integrated sensor systems deliver image and non-image based information about morphological and spectral properties of the plants. Thus, non-destructive determination of plant and plant stand parameters like plant height, tiller density, grain yield, moisture content, leaf colour, lodging and dry biomass yield become feasible but require plant and trait specific calibrations.

A first calibration and validation of plant height based on data of the LCs was established. The determined height was related to the reference values by linear regression analysis according to the model:

(3) y=b⋅x+ε

where *y* denotes the observed plant height in the field by the reference method, *x* the determined plant height, *b* the regression coefficient and *ε* the error term. Regression analysis was performed with the <regress> function of the MATLAB^®^ software package using the default settings of the function. To quantify the results the following statistics were applied: Mean relative error of calibration:

(4) $${\text{MRE}}_{C}=1/n\cdot \sum_{i=1}^{s}\left|{\hat{y}}_{i}-y_{i}\right|/y_{i}$$

where *n* denotes the number of samples in the dataset, *ŷ_i_* the determined and *y_i_* the observed plant height of plot *i* and the coefficient of correlation of calibration as the squared correlation:

(5) $${R_{C}}^{2}=r^{2}({\hat{y}}_{i},y_{i})$$

**Figure 8.**
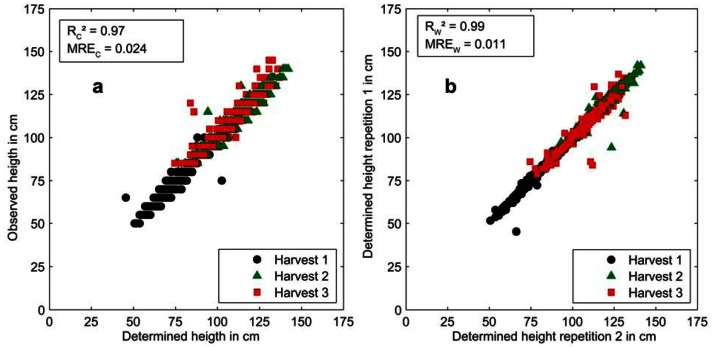
(**a**) Accuracy and (**b**) technical repeatability of plant height determination based on light curtain data of the year 2011 and 2012. *R_c_^2^* denotes the coefficient of correlation of calibration, *MRE_c_* the mean relative error of calibration, *R_w_^2^* the coefficient of correlation of repetition, *MRE_w_* the mean relative error of repetition and *Harvest* the three different timepoints of reference data collection.

The measured plant height shows a strong correlation to the reference values with *R_c_^2^* = 0.97 and *MRE_c_* = 0.024, as depicted in Figure 8(a). Besides this high accuracy, the precision of the developed plant height calibration is also very high with *R_w_^2^* = 0.99 and a *MRE_w_* = 0.011 as depicted in Figure 8(b).

### 4.4. Potential Applications of the Platform in Plant Breeding

The development of improved crop varieties is based on multi-locational field trials with thousands of genotypically distinct lines [4]. Whereas the genotyping capabilities have greatly improved in the last decade little progress has been made in the development of phenotyping methods in the last 30 years. Major limitations of field based phenotyping methods are an insufficient throughput, not totally objective results caused by manual measurements and the lack of non-destructive measurement methods for certain traits to assess the dynamic reactions of crops in response to abiotic or biotic stress.

Due to multi-sensor integration and the time- and position-specific data collection method, measurements of multiple traits like plant height, plant moisture content, tiller density and dry biomass yield become feasible with the platform based on the data collected from a single pass of the field plots. The platform enables the screening of about 250 plots per hour which results in a phenotyping capacity of multiple traits of more than 2,000 plots per day. This performance will result in a significant decrease of labor and time consumption compared to available phenotyping methods while at the same time providing more accurate and objective measurements of complex traits. Due to the non-destructiveness the phenotyping platform will open the opportunity to measure the dynamic reactions of crops in response to abiotic or biotic stress, traits that nowadays are routinely assessed only by static examinations at single time points. In addition, the different types of sensors with varying selectivities and especially the modular system architecture, allowing a flexible integration of new sensor technologies, constitute the basis for a non-destructive measurement of traits that from a technical point of view are complex to measure and not yet determinable under field conditions. Thus, more detailed information about the habitus of the plants (e.g., the angle and the size of the spikes or detailed morphological information of the leaves) or even primary and secondary metabolites may be assessed with the actual or an extended platform in routine plant breeding trials in the future opening up new avenues to crop improvement.

## 5. Conclusions and Outlook

The results of our study underline the high suitability and robustness of the developed platform for phenotyping in plant breeding trials. The system delivers high precision multi-sensor data with high spatial and temporal resolution and, due to its flexibility, provides the basis for further sensor and sensor fusion model developments for non-destructive measurements of further, even more complex agronomic traits of small grain cereals.

The modular system architecture for data collection allows a comfortable extension of the system with surplus sensor technologies like ultrasonic rangefinders, infrared thermography cameras or laser scanners. By extending the bandwidth of the main communication bus between the sensor gateways and the phenotyping server, the number of sensors can even be extended.

The first application of the platform was the non-invasive measurement of relevant traits of yield trial plots of small grain cereals, in particular triticale. Due to the modular system architecture, the platform can be adapted to different species by adapting the algorithms for data processing and the calibrations. The system architecture for data collection and processing itself can be reused without the need of any change.

The integration of the sensors into a separate module allows the adoption of the sensor systems to different carrier vehicles. The next step will be the adoption of the sensor module to mechanical interfaces of machines like high-clearance tractors which will enhance the economic value of the platform by using standard machines. In addition to a tractor pulled solution or the adoption to self-driven vehicles, even the integration into autonomous robots is possible and might be an application scenario for the future.

## Figures and Tables

**Table 1.** Detailed technical information of the different types of integrated sensor systems for the phenotyping platform (LDS: laser distance sensor; LC: light curtain; ToF: Time-of-Flight; HIS: hyperspectral imaging).

**Sensor**	**Manufacturer**	**Model**	**Supply Voltage**	**Frame rate (Hz)**	**Interface**	**Data format**
LDS-1	Leuze	ODSL 96K/V66-0-S12	24V	500	analog	Voltage
LDS-2, LDS-3	Baumer	OADM	24V	1000	analog	Voltage
LC imaging	Sitronic	Infrascan 5000	24V	170	RS485	String
3D ToF camera	Ifm	Effector 3D	24V	5	Ethernet	50 × 64 × 1 Float
HSI	EVK	Helios Core	24V	100	Ethernet	320 × 252 × 1 Byte
Colour camera	Canon	Eos 1000D	8.0V	1	USB	3.888 × 2.592 × 4 Byte compressed
Webcam	Logitec	C500	(USB)	30	USB	.ogg
GPS	Navilock	NL-402U	(USB)	1	USB	String
Rotary encoder	Wachen-dorff	WDG40A	5V	-	TTL	PWM

**Table 2.** Repeatability of processing results by light curtains (LC), laser distance sensors (LDS-1, LDS-2), 3D-Time-of-Flight cameras (3D-1, 3D-2) and hyperspectral imaging (HSI) for three different development stages of the plants from the years 2011 and 2012 based on two repetitions of 400 samples for every development stage.

**Sensor**	**Parameter**	**Harvest 1**		**Harvest 2**		**Harvest 3**	

		R_w_^2^	MRE_w_	R_w_^2^	MRE_w_	R_w_^2^	MRE_w_
**LC**	Height	0.99	0.010	0.97	0.008	0.93	0.014
	Coverage density	0.93	0.028	0.97	0.023	0.84	0.053
**LDS-1**	Height	0.93	0.031	0.83	0.028	0.85	0.029
	Penetration-depth from top	0.79	0.082	0.91	0.058	0.77	0.101
**LDS-2**	Penetration-depth from side	0.72	0.110	0.91	0.051	0.21	0.133
**3D-1**	Height	0.68	0.065	0.92	0.043	0.62	0.051
	Penetration-depth from top	0.71	0.075	0.62	0.044	0.69	0.054
**3D-2**	Penetration-depth from side	0.81	0.090	0.86	0.073	0.50	0.081
**HSI**	Coverage density	0.60	0.157	0.86	0.111	0.70	0.147
